# *In vitro* adhesion properties of Shiga toxin-producing *Escherichia coli* isolated from cattle, food, and humans

**DOI:** 10.3389/fmicb.2015.00156

**Published:** 2015-02-27

**Authors:** Nathalie Pradel, Lucie Etienne-Mesmin, Jonathan Thévenot, Charlotte Cordonnier, Stéphanie Blanquet-Diot, Valérie Livrelli

**Affiliations:** ^1^Centre de Recherche en Nutrition Humaine Auvergne, M2iSH, ‘Microbes, Intestin, Inflammation et Susceptibilité de l’Hôte’ UMR INSERM/Université d’Auvergne U1071 USC-INRA 2018, Clermont Université – Université d’AuvergneClermont-Ferrand, France; ^2^Centre de Recherche en Nutrition Humaine Auvergne, EA-4678 CIDAM, ‘Conception Ingénierie et Développement de l’Aliment et du Médicament’, Clermont Université – Université d’AuvergneClermont-Ferrand, France; ^3^Unité de Recherche M2iSH, Faculté de Pharmacie, CHU Clermont-Ferrand, Service Bactériologie Mycologie ParasitologieClermont-Ferrand, France

**Keywords:** STEC, cell lines adhesion, seropathotype, *eae*, *toxB*

## Abstract

Shiga toxin-producing *Escherichia coli* (STEC) are able to cause serious illnesses ranging from diarrhea to hemorrhagic colitis and hemolytic-uremic syndrome (HUS). These bacteria colonize the digestive tract of humans and produce Shiga-toxins, which are considered to be essential for virulence and are crucial in lethal infection. Colon colonization is supposed to be a determinant step in the development of the infection, but the virulence traits that mediate this step are unclear. We analyzed the ability of 256 STEC strains belonging to seropathotype A (the most virulent O157:H7 serotype) to seropathotype E (not involved in human disease) to adhere to HEp-2, HCT-8, and T84 cell lines. Of the 256 STEC tested most (82%) were non-adherent in our assays. The adhesion levels were globally low and were not related to pathogenicity, although the highest levels were associated to O26:H11 and O103:H2 strains of seropathotype B (associated with HUS but less commonly than serotype O157:H7), possessing both the *eae* and *toxB* genes.

## INTRODUCTION

Shiga toxin (Stx)-producing *Escherichia coli* (STEC) can be associated with human diseases, ranging from uncomplicated diarrhea to hemorrhagic colitis (HC) and hemolytic-uremic syndrome (HUS; Hussein, 2007). Enterohemorrhagic *E. coli* (EHEC) constitute a subset of pathogenic STEC that also contain the LEE (Clements et al., 2012). The most prominent serotype within the EHEC group is O157:H7 (Paton and Paton, 1998). STEC food-borne infections, either outbreaks or sporadic cases, appear worldwide.

As example, an outbreak with 1000s cases of food-borne illness has been caused by an emerging atypical O104:H4 Stx-producing pathogen in 2011 in Germany (Bielaszewska et al., 2011; Frank et al., 2011; Clements et al., 2012; Trachtman et al., 2012). Several studies have shown a high prevalence of STEC belonging to a wide range of serotypes in animals and food products (Pradel et al., 2000; Beutin, 2006; Hussein, 2007). However, only a limited number of serotypes have been associated with human disease, among which O157:H7 is predominant (Rangel et al., 2005). The association of serotypes with disease of varying severity in humans, and with outbreaks or sporadic disease, has led to the proposal that STEC be classified into five seropathotypes: A (the most virulent O157:H7 serotype), B (associated with outbreaks and HUS but less commonly than serotype O157:H7), C (associated with sporadic HUS but not typically with outbreaks), D (associated with diarrhea but not with outbreaks or HUS), and E (serotypes that have not been involved in disease in humans; Karmali et al., 2003). STEC colonize the digestive tract of humans and produce Stx1 and/or Stx2 which are essential for virulence. Different combinations of potential pathogenicity factors have been observed in clinical isolates, in addition to the production of Stx (Paton and Paton, 1998; Girardeau et al., 2005; Pradel et al., 2008). However, the known virulence factors do not allow differentiation of STEC strains with a high pathogenic potential from their counterparts of lesser clinical significance.

The terminal ileum and the colon are supposed to be the main sites of EHEC colonization in humans, whereas in cattle, the terminal recto-anal junction is thought to be the primary site of colonization of the bovine gastrointestinal tract (Naylor et al., 2003; Chong et al., 2007; Mahajan et al., 2009). Some STEC strains adhere to epithelial cells in a histopathological lesion pattern known as attaching and effacing (A/E) adhesion, first described in enteropathogenic *E. coli* (EPEC), characterized by the effacement of microvilli and the formation of actin pedestals, and involving intimin EaeA (Hayward et al., 2006). *In vitro* adherence of STEC strains has been examined using different epithelial cell lines under a range of experimental conditions. The “gold standard” method was originally described to analyze virulence properties of *E. coli* strains involved in diarrhea (Knutton et al., 1989). The HEp-2 or HCT-8 cell assay is considered as a suitable model to study A/E lesions in STEC (McKee and O’Brien, 1995). The human intestinal T84 cell line is also used for studies of the adhesion properties since these cells resemble human colonic epithelial cells, which are thought to be the *in vivo* target cells for STEC infection (Winsor et al., 1992; Li et al., 1999). However, not all the pathogenic STEC strains are able to adhere in an A/E mechanism. Thus, the virulence traits that mediate STEC colonization are not fully understood.

In this study, the adhesion properties of a collection of 256 STEC strains (belonging to seropathotypes A to E), isolated from humans, foods, or animals, were analyzed using *in vitro* adhesion tests to human epithelial cell lines (HEp-2, HCT-8, T84) and relationships between adhesion, virulence factors, and pathogenicity were investigated.

## MATERIALS AND METHODS

### BACTERIAL STRAINS

The distribution according to origin and seropathotype of the 256 STEC isolates tested in this study is given in **Table 1**. Among the 256 STEC strains, 32 were isolated from patients suffering from HUS or HC, 187 were isolated from bovine feces, 26 from food samples, and 11 from asymptomatic children (Pradel et al., 2000, 2002). EHEC O157:H7 strains EDL933 (ATCC 43895) and 86-24 (Tarr et al., 1989), and EPEC strain E2348/69 were used as references strains. *E. coli* strain K-12 C600 was used as a negative control. Bacteria were stored in Luria broth (LB), with 10% glycerol at -80°C. Unless otherwise stated, the strains were grown overnight at 37°C in LB.

**Table 1:** Number of strains analyzed in this study according to origin and seropathotype.

		Seropathotypes						
		A	B	C	D	E	ND	Total
	Number of serotypes	1	2	19	22	49	–	93
Origin	Patients (HUS, HC)	10	8	10	0	0	4	32
	Bovine feces	1	2	57	37	79	11	187
	Food	2	0	6	4	11	3	26
	Asymptomatic children	0	2	0	9	0	0	11

	Total	13	12	73	50	90	18	256

*HUS, hemolytic-uremic syndrome; HC, hemorrhagic colitis; ND, not determined*.

### CELL LINE AND CELL CULTURE

The human laryngeal epithelioma cell line HEp-2 (ATCC CCL-23; American Type Culture Collection, USA), the human ileocecal carcinoma cell line HCT-8 (ATCC CCL-244), and the human colonic carcinoma cell line T84 (ATCC CCL-248) were used for investigating STEC adhesion profiles. The three cell lines were grown with DMEM supplemented with 10% fetal bovine serum (FBS; Lonza) and antibiotics (2% penicillin G-streptomycin–amphotericin B) at 37°C, in an atmosphere of 5% CO_2_. The T84 cells were differentiated by growing five days in 24-well plates coated with collagen I (Roche). About 10^6^ viable cells were used for each bacterial infection in 24-well plates.

### ADHESION ASSAYS

The bacterial strains were grown for 3 h (exponential growth phase) or 18 h (late growth phase) at 37°C before infection, in LB or in Cell Culture Medium (CCM) with 50% HAM-50% DMEM. Bacteria were harvested by centrifugation at 7000 ×*g* for 10 min and suspended in CCM in the presence of 1% α-D-mannoside to a final concentration of 10^8^ CFU per ml, which was checked by plating on LB agar for each inoculum. The culture cells were washed three times with PBS pH 7.4 and then infected with 10^8^ bacteria for 3 h at 37°C at a multiplicity of infection (MOI) of 100. After 3 h, non-adherent (NA) bacteria were removed from the cells by three washes with PBS. Bacterial adherence patterns were examined with phase-contrast microscopy after Giemsa staining. For quantitative adherence assays, the cells were gently scraped off with 1% triton X-100 (Sigma) in PBS, and serial 10-fold dilutions were plated overnight at 37°C onto Luria agar plates. Each experiment was repeated at least three times. Variations in the level of F-actin in HEp-2 and HCT-8 cells were estimated by fluorescence actin staining (FAS) assay (Knutton et al., 1989). Scanning electron microscopy (SEM) was performed as previously described by Xicohtencatl-Cortes et al. (2007).

### DETECTION OF GENES ASSOCIATED WITH ADHESION

All STEC isolates were analyzed for the presence of genes encoding for EaeA, EfaI, ToxB, LpfA, Saa, East1, EspP, and Iha. The corresponding *eae*, *efa1*, *toxB, lpf, saa*, *astA, espP*, and *iha* genes were amplified using primers listed in **Table 2**. The presence of the genes was determined directly by PCR and/or by colony blot hybridization using probes labeled with α-^32^P-dCTP, as described previously (Pradel et al., 2002).

**Table 2:** Primers used for genes targeting and proportion of positive strains.

Target Gene	Primer name	Reference	Oligonucleotide sequence (5′ to 3′)	Number of positive strains (out of 256 strains)
*eae*	*eae*F3	Pradel et al. (2008)	GAACGGCAGAGGTTAATCTGC	38 (15%)
	*eae*R3		TCAATGAAGACGTTATAGCCC	
*efa*1	88AT	Nicholls et al. (2000)	AAGGTGTTACAGAGATTA	28 (11%)
	88TN		TGAGGCGGCAGGATAGTT	
*toxB*	Tox1	Paton and Paton (1998)	ATCCTGAAACAAAACGAAACG	27 (10%)
	Tox2		AATTGTATGCTCTAGACTCC	
*lpfA*_O113_	*lpfA*-F	Doughty et al. (2002)	ATGAAGCGTAATATTATAG	201 (79%)
	*lpfA*-R		TTATTTCTTATATTCGAC	
*lpfA*_O157/OI-141_	*lpf*O141-F	Szalo et al. (2002)	CTGCGCATTGCCGTAAC	
	*lpf*O141-R		ATTTACAGGCGAGATCGTG	
*lpfA*_O157/OI-154_	O154-FCT	Toma et al. (2004)	GCAGGTCACCTACAGGCGGC	
	O154-RCT		CTGCGAGTCGGCGTTAGCTG	
*saa*	*Saa*DF	Nicholls et al. (2000)	CGTGATGAACAGGCTATTGC	82 (32%)
	*Saa*DR		ATGGACATGCCTGTGGCAAC	
*ast*A	*east*11a	Yamamoto et al. (1997)	CCATCAACACAGTATATCCGA	20 (8%)
	*east*11b		GGTCGCGAGTGACGGCTTTGT	
*espP*	*esp*A	Pradel et al. (2008)	AAACAGCAGGCACTTGAACG	122 (48%)
	*esp*B		GGAGTCGTCAGTCAGTAGAT	
*iha*	*iha*-F	Tarr et al. (2000)	CTGGCATGCCGAGGCAGTGC	169 (66%)
	*iha*-R		CGTTGCCACTGTTCCGCCAGG	

### STATISTICAL ANALYSIS

The data were analyzed with Epi Info version 6.02 by the χ^2^ test, except for the variable needing a two-tailed Fischer exact test. The Student *t*-test for unpaired data was used at the 5% level of significance for the comparison of values.

## RESULTS

### QUANTITATIVE ADHESION OF 17 STEC STRAINS TO THE T84 CELL LINE

**FIGURE 1:**
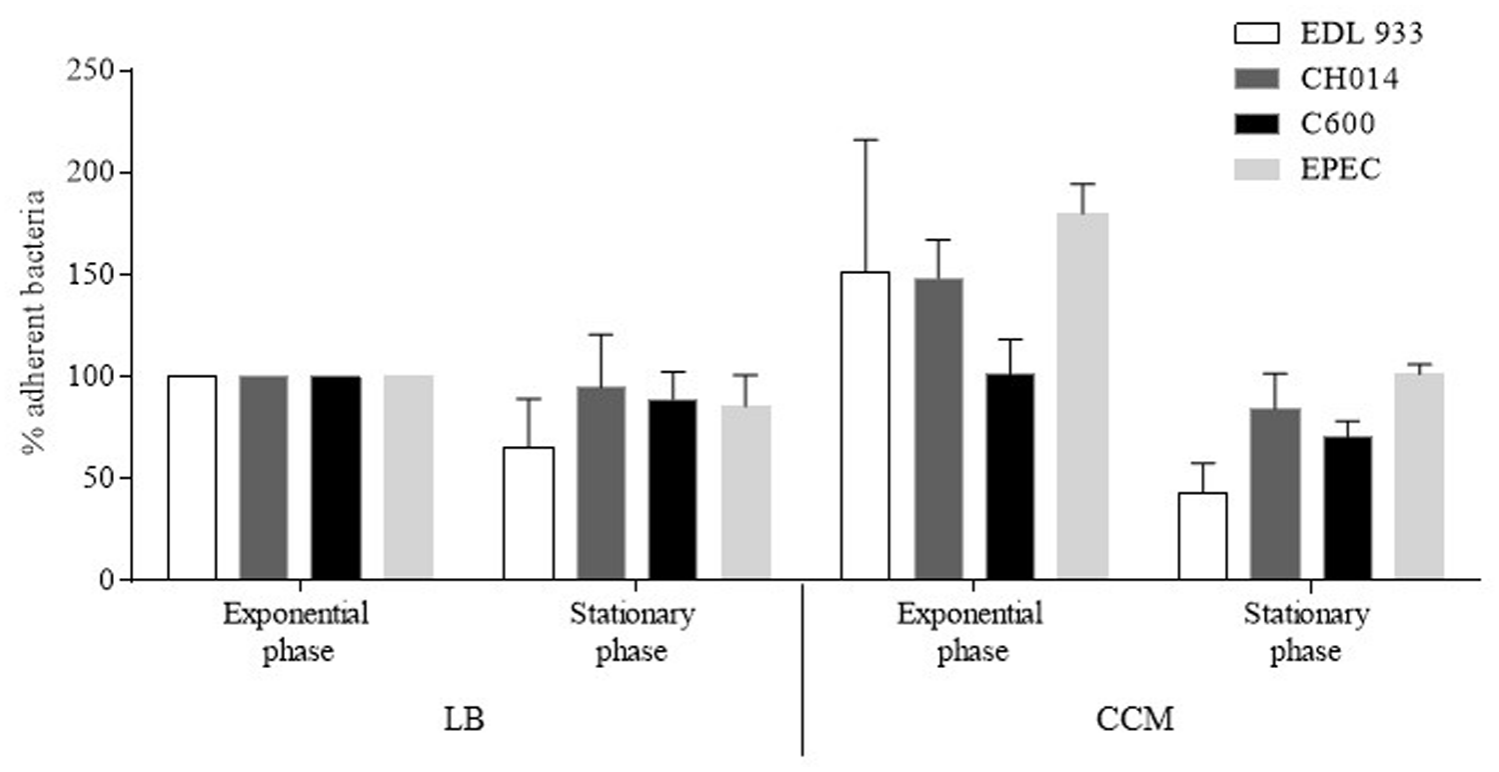
**Determination of experimental conditions for bacterial adherence of *Escherichia coli* strains to human colonic carcinoma T84 cells.** Cells were infected with bacteria grown for 3 h (exponential growth phase) or 18 h (stationary growth phase), in Luria Bertani Broth (LB) or in Cell Culture Medium (CCM), at 37°C before infection. The cells were then washed, treated with Triton X-100, and the recovered adherent bacteria were diluted and plated for colony forming unit counting. Results are expressed as percentage of adherent bacteria, relative to adherent bacteria obtained after a 3 h growth in LB taken as 100%. EDL933: EHEC O157:H7; CH014: non-O157 STEC; C600: K-12 *E. coli*; E2348/69: EPEC. All assays were performed independently at least three times. Results are means ± SEM of bacteria adhering to T84 cells.

**FIGURE 2:**
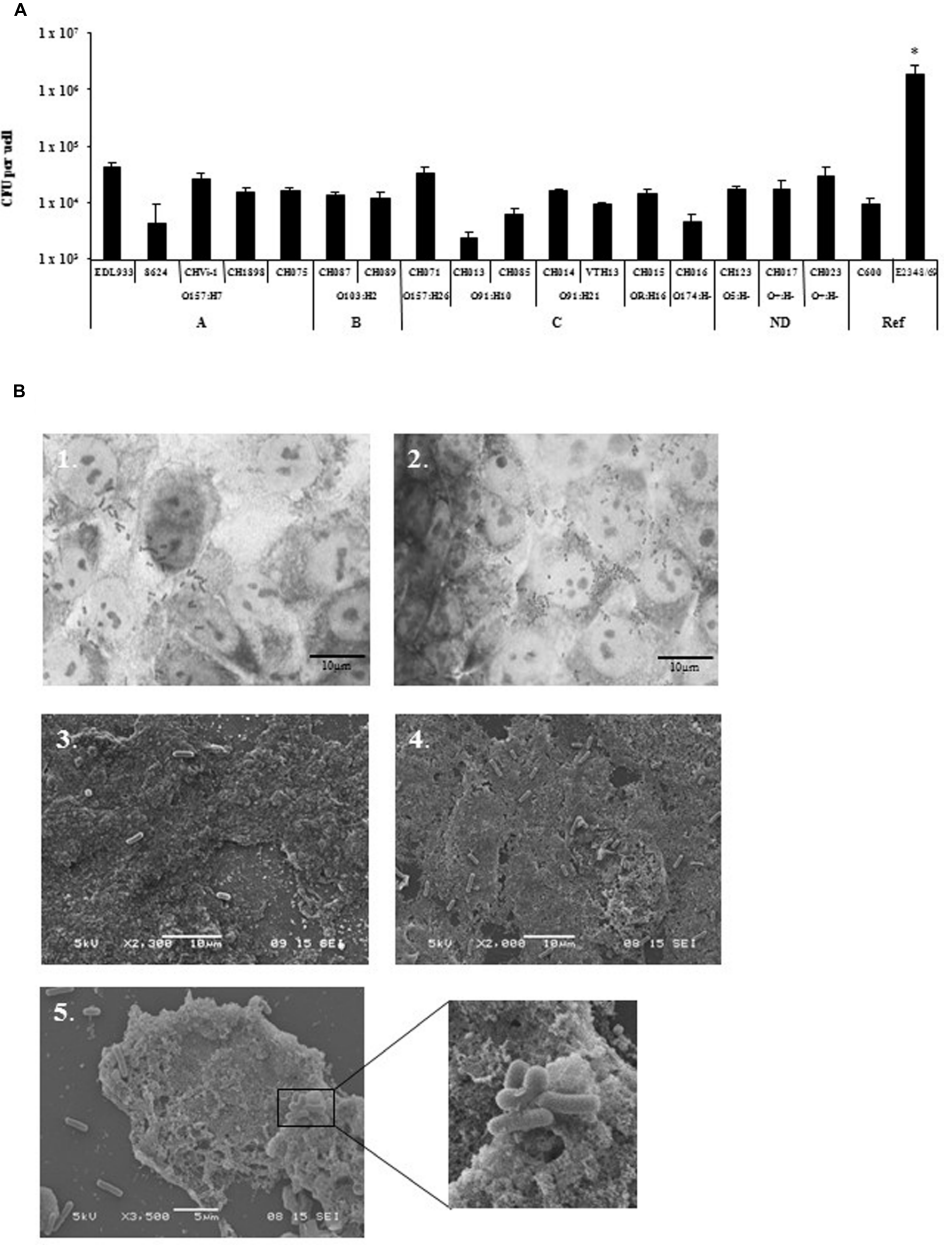
**Adhesion of STEC strains to T84 cells line. (A)** Quantitative adhesion of 17 STEC strains on the human colonic carcinoma cell line T84. Strains were grown to exponential growth phase at 37°C in CCM before infection. All assays were performed independently at least three times. Results are means ± SD of bacteria adhering to T84 cells in CFU/well for the replicate experiments. A, seropathotype A; B, seropathotype B; C, seropathotype C; ND, seropathotype not determined; Ref, reference strains. *The level of adhesion of EPEC E2348/69 strain was significantly different from all other strains tested (*p* < 0.01).** (B)** Adhesion pattern of EHEC O157:H7 (EDL933) strain (1, 3) and EPEC (E2348/69) strain (2, 4, and 5) stained with Giemsa (1, 2), or after scanning electron microscopy (SEM) analysis (3, 4, and 5). Scale bar = 10 μm for Giemsa and SEM, Magnification ×2,300 (3), ×2,000 (4), ×3,500 (5) and x10,000 (enlarged box).

Different bacterial growth phases (exponential growth phase vs. late-log phase), and types of bacterial culture media (LB vs. CCM) were tested in preliminary infection experiments on the T84 human colonic carcinoma cell line. These assays were performed with two virulent STEC strains (O157:H7 strain EDL933 and O91:H21 strain CH014), the EPEC reference strain (E2348/69) as a positive control, and the *E. coli* strain K-12 C600 as a negative control (**Figure 1**). The best adhesion levels were obtained with strains grown for 3 h in CCM. For further quantitative experiments, we thus chose to incubate the bacteria for 3 h at 37°C in CCM before infection. Quantitative experiments, performed on 17 STEC strains isolated from human cases, indicated that the number of bacteria adhering to the T84 cells was between 2.4 × 10^3^ and 4.4 × 10^5^ CFU/well (**Figure 2A**). The number reached 2 × 10^6^ CFU/well for the EPEC reference strain, indicating that STEC adhered to very low levels compared to the EPEC strain, and just above the level of the negative control C600. Adherence patterns were investigated after staining with Giemsa and by SEM observations. The STEC strains adhered sparsely, whereas the EPEC formed micro-colonies (**Figure 2B**).

### ADHESION PROPERTIES OF 256 STEC TO THE HCT-8 AND HEP-2 CELL LINES

**FIGURE 3:**
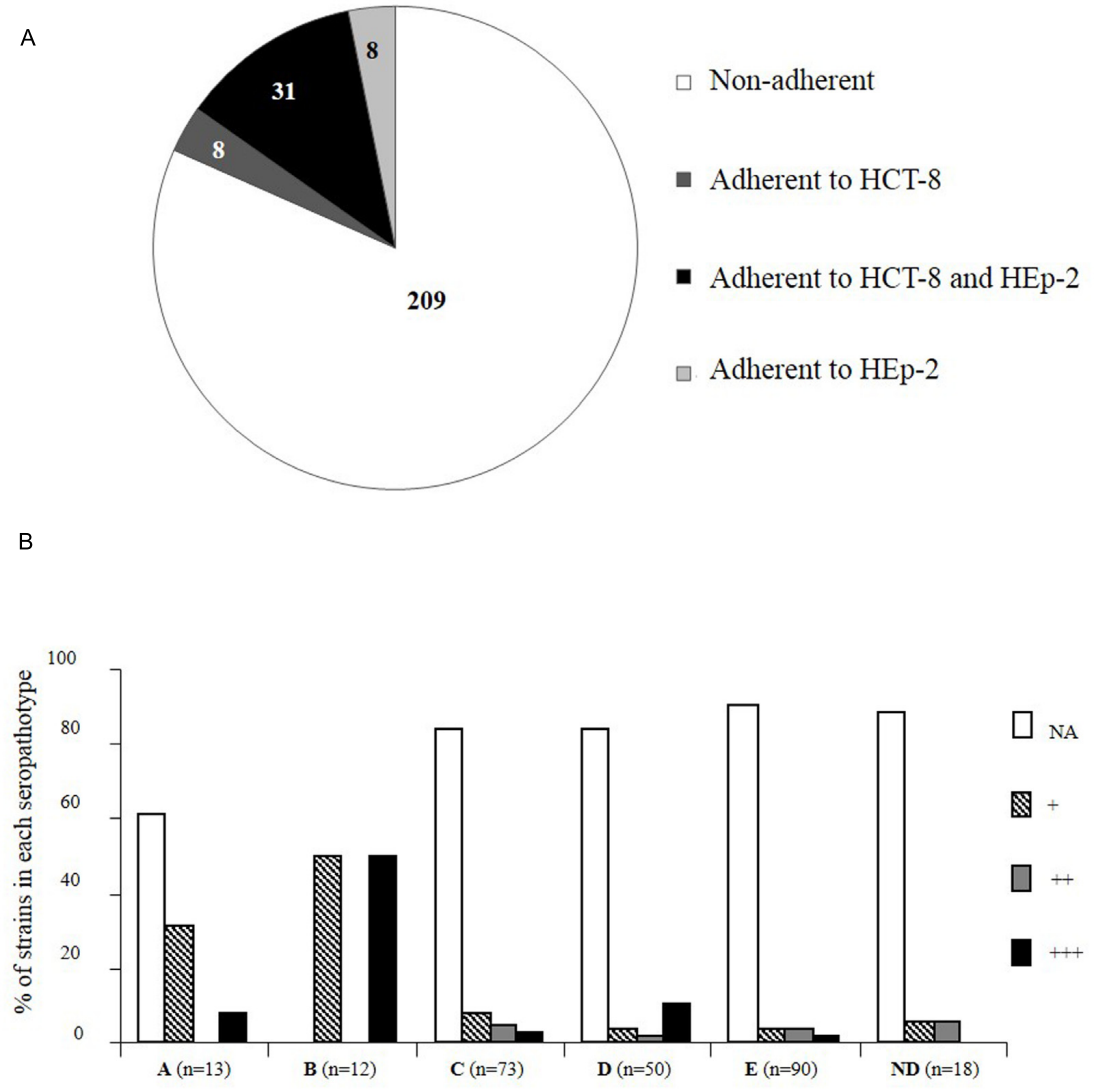
**Distribution of Shiga-toxin producing *E. coli* strains according to adhesion properties to HCT-8 and/or HEp-2 cell lines and seropathotype. (A)** Number of STEC strains adherent to HCT-8 and/or HEp-2 cells or non-adherent (NA; less than 1 bacterium per cell).** (B)** Results are expressed as the percentage of strains that adhered or not, and the level of adhesion, in each seropathotype. +, 1 to 5 bacteria/cell; ++, 5 to 20 bacteria/cell; +++, more than 20 bacteria/cell. The data were analyzed with Epi Info version 6.02 by the Fischer exact test. Groups were established by comparison with the adhesion level of the positively adhering EPEC (E2348/69) and the negatively adhering K-12 *E. coli* (C600) control strains. ND, seropathotype not determined.

Since very low levels of adhesion were observed with the pathogenic STEC tested on T84 cells, we chose to analyze the 17 STEC strains and 239 additional STEC strains of diverse origins on two cell lines commonly used for *in vitro* adhesion assays: HCT-8 (from human ileocecal carcinoma), and HEp-2 (from human larynx carcinoma) cell lines (**Figure 3**). We considered as adherent the strains adhering to at least one of the two cell lines. Adhesion to HCT-8 or HEp-2 showed few strains efficiently adhering over a 3 h course of infection: among the 256 STEC strains, 209 (82%) were considered as NA; (i.e., less than 1 bacterium per cell; **Figure 3A**). Only 18% (47/256) of the strains adhered to at least one of the cell lines. Among them, 31 adhered both to HEp-2 and to HCT-8.

The great majority of STEC strains were NA whatever the seropathotype (A: 61% NA, C: 84% NA, D: 84% NA, E: 91% NA), except for the seropathotype B for which all the strains tested were adherent (**Figure 3B**). The adherent strains belonging to seropathotype A were associated to a low level of adhesion (+, 1 to 5 bacteria per cell; Fisher, *P* = 0.01), and the seropathotype B was associated to the higher level of adhesion (+++, more than 20 bacteria per cell; Fisher, *P* = 0.002; **Figure 3B**; **Table 3**). Indeed, among the 39 STEC adhering to HCT-8, 15 presented an enhanced adherence (≥ 20 bacteria per cell), including six of seropathotype B (four O103:H2 and two O26:H11), and one of seropathotype A (the O157:H7 strain isolated from bovine feces). Nine strains exhibited an enhanced adherence (≥20 bacteria per cell) to HEp-2 cells. These nine adhering strains were all non-O157:H7 and included two O103:H2 strains of seropathotype B isolated from bovine feces.

**Table 3:** Properties of the 47 adherent STEC and of the corresponding 39 NA STEC of identical serotypes.

Sero- pathotype	Serotype	Number of strains	Source	HCT-8^§^	HEp-2^§^	FAS assay^||^	Presence or absence^†^ of adhesion									
							*eae*^‡^	*efaI*	*toxB*	*lpf_O157_*	*lpf_O113_*	*lpf_O91_*	*saa*	*astA*	*espP*	*iha*
A	O157:H7*	7	Human	0	0	-	γ	+	+	+	-	-	-	-	+	+
		3	Human	2	2	+ (1)	γ	+	+	+	-	-	-	-	+	+
		1	Animal	50	0	+/-	γ	+	+	+	-	-	-	-	+	+
		1	Food (meat)	2	2	+	γ	+	+	+	-	-	-	-	+	+
		1	Food (meat)	0	0	-	γ	+	+	+	-	-	-	-	+	+

B	O103:H2	2	Animal	35	30	+	ε	+	+	-	-	-	-	-	+	+
		2	Human	30	3	+	ε	+	+	-	-	-	-	-	+	+
		6	Human	5	5	+	ε	+	+	-	-	-	-	-	+	+
	O26:H11	2	Human	30	10	+	β	+	+	-	+	+	-	-	+	+

C	O157:H26	1	Human	4	4	+	β	-	-	-	-	-	-	-	-	ND
	O174:H2	1	Food (meat)	0	5	-	-	-	-	-	+	+	-	-	+	-
		1	Food (cheese)	0	0	-	-	-	-	-	+	+	+	-	+	-
		3	Animal	10	18	-	-	-	-	-	+	+	+	-	+	-
		2	Animal	5	0	-	-	-	-	-	+	+	+	-	+	-
		6	Animal	0	0	-	-	-	-	-	+	+	+	-	+	-
	O5:H-	1	Human	0	4	-	β	+	-	-	+	+	-	-	+	ND
	O6:H4	1	Human	10	8	-	-	-	-	-	-	-	-	-	-	ND
	O8:H2	1	Human	0	4	-	-	-	-	-	+	+	-	-	-	ND
	O91:H21	1	Food (cheese)	30	10	-	-	-	-	-	+	+	+	-	-	-
		2	Food (meat)	0	0	-	-	-	-	-	+	+	+	-	-	-
		3	Animal	0	0	-	-	-	-	-	+	+	+	-	-	-
		2	Human	0	0	-	-	-	-	-	+	+	+	-	+	-
	O98:H-	1	Animal	0	0	-	γ	-	-	-	+	+	-	-	+	ND
	OR:H16	1	Human	20	35	-	-	-	-	-	+	+	+	-	+	-

D	O112ac:H19	1	Human	40	0	-	-	-	-	-	+	+	-	-	-	ND
		2	Animal	0	0	-	-	-	-	-	+	+	+ (1)	-	+ (1)	ND
	O113:H4	7	Animal	0	0	-	-	-	-	-	-	-	-	+ (1)	-	+
		1	Food (meat)	0	2	-	-	-	-	-	-	-	-	-	-	+
		1	Food (meat)	0	0	-	-	-	-	-	-	-	-	-	-	+
	O127:H+	1	Human	30	20	+	γ	-	-	-	-	-	+	-	+	+
	O171:H2	1	Animal	5	0	-	-	-	-	-	+	+	+	-	+	+
		6	Animal	0	0	-	-	-	-	-	+	+	-	-	-	+
	O75:H8	1	Food (meat)	30	10	-	-	-	-	-	+	+	-	-	-	ND
	O77:H18	1	Animal	50	60	-	-	-	-	-	+	-	+	-	+	ND
	OR:H2	1	Animal	10	2	-	-	-	-	-	+	+	-	+	-	ND
	OR:K1:H-	1	Human	30	50	-	-	-	-	-	+	+	-	-	-	ND

E	“O”K84:H19	1	Animal	15	25	-	-	-	-	-	+	+	-	-	-	ND
	O102:H21	1	Animal	10	0	-	-	-	-	-	+	+	-	-	-	-
	O103:H14	1	Food (cheese)	30	3	-	-	-	-	-	+	+	-	-	-	-
	O150:H8	1	Food (meat)	0	5	-	-	-	-	-	+	+	-	-	-	ND
	O153:H8	1	Food (meat)	0	20	-	-	-	-	-	+	+	+	+	+	+
	O15:H16	2	Animal	15	0	-	-	-	-	-	+	+	-	+	+ (1)	ND
	O54:H2	1	Food (meat)	0	3	-	-	-	-	-	+	+	-	-	-	ND

ND	OR:H+	1	Animal	10	20	-	ζ	-	-	-	+	-	-	-	+	-
	O+:H-	1	Human	0	2	-	ε	+	-	-	+	+	-	-	+	ND

EPEC	O127:H6	1	E2348/69	50	40	+	α	+	-	-	-	-	-	-	-	-
C600	K12	1	Laboratory	0	0	-	-	-	-	-	-	-	-	-	-	-

*EDL933 and 86-24 reference strains are included. **^§^** Mean number of adherent bacteria /HCT-8 and /HEp-2 cells. ^∣∣^FAS activity on HEp-2/HCT-8 cells: +, FAS-positive on HCT-8 and HEp-2; -, FAS-negative on HCT-8 and HEp-2; +/-, FAS-positive on HCT-8 only; number between parentheses indicates number of FAS-positive strains. ^†^+, found positive by PCR and/or probe staining for the corresponding gene; -, found negative by PCR and/or probe staining for the corresponding gene; number between parentheses indicates number of positive strains. ^‡^*eae* variant identified by PCR (Pradel et al., 2000). EPEC, enteropathogenic *Escherichia coli*; C600, *E. coli* K12 C600 negative control. ND, not determined.

### ASSOCIATION BETWEEN ADHESION PROPERTIES AND GENES ENCODING FOR ADHESION FACTORS

Data regarding the presence of genes *eae*, *efa1*, *toxB*, *lpf*, *saa*, *astA*, *espP*, and *iha* are given in **Tables 2** and **3**. Among the 256 STEC strains, 38 (15%) were *eae*-positive (possessed the gene encoding for intimin), including all the STEC belonging to the seropathotypes A (13) and B (12). Twenty-two of these adhered either to HCT-8 and/or to HEp-2 cells, but most (13 of 22) adhered at a low level (1 to 5 bacteria/cell), and only 17 were clearly FAS-positive by the fluorescent actin staining assay. Overall, the presence of *eae* was associated with adhesion (Fisher, *P* < 0.00000001), and a strong association was observed between FAS-positive isolates and belonging to seropathotype B (Fischer, *P* < 0.0000001), or isolation from a human with or without disease (Fischer, *P* = 0.00002). The gene *efa1*, associated to the *eae* gene and encoding for EHEC factor for adherence, was detected in 28 *eae*-positive strains (11% of the STEC).

Only 27 of 256 STEC (10%) were *toxB* positive: 17 adhered to HEp-2 and/or HCT-8 cells, and belonged to A or B seropathotype. An association was established between the *toxB* gene and adhesion (Fisher, *P* < 0.00000001).

Among the 256 strains, 201 (79%) were positive for the *lpf* gene encoding long polar fimbriae subunit A. Thirty three of the 47 adherent strains were positive for *lpf_O157_*, *lpf_O113_,* or *lpf_O91_*. operon. Regarding *saa,* encoding the STEC autoagglutinating adhesin, 82 strains (32%), belonging to the less virulent seropathotypes C, D, E, were positive, of which 11 were adherent. Regarding *astA*, encoding the East1 enteroaggregative heat-stable toxin 1, only 20 of the 256 STEC (8%) belonging to the seropathotypes D and E were positive, including 3 adherent isolates. Among the 256 strains, 122 (48%) were positive for the autotransporter EspP, shown to be involved in the formation of macroscopic rope-like fibers (Xicohtencatl-Cortes et al., 2010). Thirty-one adhered to HEp-2 and/or HCT-8 cells. Finally, 169 strains (66%) were positive for the gene encoding for Iha (a protein that confers adherence similar to *Vibrio cholerae* IrgA), of which 21 were adherent. No association was established between adhesion or seropathotype and the presence of the *lpf*, *saa, astA*, *efa1*, *espP*, or *iha* genes.

## DISCUSSION

Shiga toxin-producing *E. coli* strains are an important cause of diarrheal and renal diseases in humans. They are associated with colonic pathology, and are considered as colonic pathogens, but the intestinal colonization process is still not well defined. In the absence of a suitable animal model for STEC infection that reproduces the typical human disease from the initial step of colonization to HUS (Mallick et al., 2012), adherence to epithelial cell lines was expected to mimic the *in vivo* situation. In this study, we analyzed the adhesion properties of a collection of 256 STEC strains isolated from cattle, food, and humans, using the intestinal epithelial cell lines HCT-8 (from human ileocecal carcinoma), T84 (from human colonic carcinoma), and the HEp-2 cells from human larynx carcinoma. More than half (51%) of the strains of human origin (including patients and asymptomatic children) were adherent, while one third (34%) of the STEC isolated from food, and only 8% of the cattle strains were found to adhere. The strains belonged to the five seropathotypes A (most virulent) to E (not involved in human disease), described according to disease severity in humans, and to outbreaks or sporadic diseases (Karmali et al., 2003). Among the 256 STEC, the seropathotype B was significantly associated with the highest adherent strains on the HEp-2 or HCT-8 models. Our study revealed that, among the 47 adhering strains, 16 adhered only to HEp-2 or HCT-8, indicating that HEp-2 cells, widely used to explore adhesion of intestinal pathogenic *E. coli* might lack specific receptors. On the T84 model, EHEC O157:H7 strains (A seropathotype) adhered significantly more than those belonging to other serotypes, but the levels of adhesion observed were low. Interestingly, a high level of adhesion was not always associated with pathogenicity (assessed by the seropathotype): among the 18 STEC exhibiting the highest level of adhesion to HEp-2 and/or HCT-8 cells, eight belonged to the seropathotypes D and E. It would be interesting to test the highly adherent strains in a suitable *in vivo* model to determine their virulence, since they might represent emerging pathogens. The O104:H4 outbreak in Germany in 2011 highlighted the danger of such emerging pathogens, the O104:H4 strain possessed enteroaggregative adhesion factors that made it particularly virulent.

Studies evaluating the survival of an *E. coli* O157:H7 strain in simulated *in vitro* models of human digestive tract revealed bacterial growth and multiplication in the distal parts of the small intestine, and a progressive elimination of the pathogen in the colon (Etienne-Mesmin et al., 2011b; Thevenot et al., 2013). In human and bovine small intestinal xenografts, a large number of EHEC was also detected in the small intestinal lumen, but bacteria did not attach to the epithelium (Golan et al., 2011). The same study revealed poor bacterial growth associated with the formation of A/E lesions in human colon xenografts (Golan et al., 2011). In this study, we observed low levels of adhesion on colonic derived cells, in accordance with studies conducted using human intestinal IVOC, where a preferential tropism of EHEC for the follicle-associated epithelium (FAE) overlying ileal Peyer’s patches (PPs), associated with a lack of colonic adhesion has been shown (Phillips et al., 2000; Fitzhenry et al., 2002; Chong et al., 2007). The PPs-rich distal ileum may represent the initial site of EHEC adhesion, from where bacteria would either spread to other regions of the gut, or translocate through M cells to underlying macrophages, which may be the first step in EHEC translocation and subsequent toxin transport across the intestinal barrier (Etienne-Mesmin et al., 2011a).

Several surface proteins and fimbriae structures have been described as putative adherence factors in STEC (Nicholls et al., 2000; Tarr et al., 2000; Fitzhenry et al., 2002, 2006; Toma et al., 2004; Cergole-Novella et al., 2007; Torres et al., 2007; Mahajan et al., 2009; Xicohtencatl-Cortes et al., 2009, 2010). Among them, intimin EaeA might play a role in tissue tropism, with intimin gamma appearing to restrict colonization of O157:H7 strains to human FAE (Fitzhenry et al., 2002). In our study, in addition to the *eae* gene, the *toxB* gene was clearly associated with adhesion to HEp-2, HCT-8, and T84 cell lines and isolation from humans. Taken together, adhesion levels were globally low among the 256 STEC tested and the *eae*^+^*toxB*^+^ STEC of seropathotype B presented the highest adhesion level. The host and tissue specificity might also involve several other bacterial and host determinants important *in vivo* (Mundy et al., 2007; Ho et al., 2008; Xicohtencatl-Cortes et al., 2009). As previously reported for the *lpf* operons, their expression could be controlled by a tightly regulated process, depending on gastrointestinal conditions such as bile salts (Torres et al., 2007; Chassaing et al., 2013), rendering the role of Lpf difficult to establish during binding to epithelial cells.

Taken together, our data showed that it is very difficult to assess STEC virulence and ability to cause outbreaks in these models, and that *in vitro* adherence may not be an accurate reflection of molecular interactions that occur between STEC and human colonic epithelium *in vivo*. Developing more appropriate models to study the mechanisms of the first colonization steps would help in designing novel therapeutic approaches to EHEC infection.

## AUTHOR CONTRIBUTIONS

Conceived and designed the experiments: NP, LEM, VL. Performed the experiments: NP, LEM, JT, CC. Analyzed the data: NP, LEM, VL. Contributed reagents/materials/analysis tools: SBD, VL. Wrote the paper: NP, LEM, VL.

## Conflict of Interest Statement

The authors declare that the research was conducted in the absence of any commercial or financial relationships that could be construed as a potential conflict of interest.
